# Early administration of a single dose of parenteral estrogen decreases inflammation in the heart for 45 days after severe burns

**DOI:** 10.1186/cc12227

**Published:** 2013-03-19

**Authors:** PE Pepe, JG Wigginton, JW Gatson, AH Idris

**Affiliations:** 1 University of Texas Southwestern Medical Center, Dallas, TX, USA

## Introduction

Patients with severe burn injury experience a rapid elevation in multiple circulating proinflammatory cytokines, with the levels correlating with both injury severity and outcome. In animal models, accumulations of these cytokines have been observed in remote organs, including the heart, brain and lungs. However, data are lacking regarding the long-term levels of cytokines in the heart following severe burn injury and also how infusion of parenteral estrogen, a powerful anti-inflammatory agent, would affect these levels. Using a rat model, we studied the effects of a full-thickness third-degree burn on cardiac levels of IL-6 and TNFα over 45 days with and without 17β-estradiol infusion.

## Methods

A total of 168 male rats were assigned randomly to one of three groups: (1) Sham burn (no actual injury) group (*n = *8); (2) Placebo (no treatment) burn group (*n = *80); and (3) E2 (estrogen treatment) burn group (*n = *80). Groups 2 and 3 had 40% TBSA third-degree dorsal burns, early fluid resuscitation and, 15 minutes post burn, 0.5 mg/kg intraperitoneal estrogen (Group 3) or placebo (Group 2). From each group of 80, eight animals were sequentially sacrificed (and cardiac tissue was sampled for IL-6, TNFα, IL-1β) at one of 10 respective time points: 0, 0.5, 1, 2, 4, 6, 8, 18, 24 hours and day 7 post burn (at day 7 only for the eight shams). The markers were measured by ELISA method.

## Results

In the burned rats, 17β-estradiol significantly decreased the cardiac levels of TNFα at all time points through 45 days post burn, with the sham animal levels (30 pg/mg) more comparable with the estradiol group (70 pg/mg), and significantly less than the placebo animals (332 pg/mg). Similarly, IL-6 levels in the sham animals (70 pg/mg) were comparable with the estradiol group (86.5 pg/mg), and significantly less than the placebo group (730 pg/mg), even at 45 days post burn.

## Conclusion

Following severe burn injury in an animal model, an early single dose of estrogen can decrease the prolonged let alone the early onset of cardiac inflammation. Based on these data, clinical studies of estrogen infusions should be seriously entertained as estrogen may not only be an inexpensive, simple adjunctive therapy in burn management, it may also obviate the need for many subsequent interventions altogether and even diminish mortality.

